# Training Healthcare Professionals on How to Promote Physical Activity in the UK: A Scoping Review of Current Trends and Future Opportunities

**DOI:** 10.3390/ijerph18136701

**Published:** 2021-06-22

**Authors:** Jake Netherway, Brett Smith, Javier Monforte

**Affiliations:** Department of Sport and Exercise Sciences, Durham University, Durham DH1 3HN, UK; jake.r.netherway@durham.ac.uk (J.N.); javier.monforte@durham.ac.uk (J.M.)

**Keywords:** physical activity, health promotion, training, education, healthcare professionals, continuing professional development, undergraduate education, E-learning

## Abstract

What physical activity (PA) training do current and future healthcare professionals (HCPs) receive in the UK? How is PA training delivered to them? The present scoping review looks at existing evidence to respond these questions. Seven databases were searched: Medline, SportDISCUS, PsycINFO, EMBASE, CINAHL, Cochrane Review Database, and Web of Science. Grey literature sources and key stakeholders were consulted. Studies were screened for inclusion, data were extracted and charted, and findings were synthesised according to the two research questions. Of the 3535 identified studies, 25 were included. The results show that no standardised approach was used to deliver PA promotion in HCPs training. PA training content was chiefly underpinned by an epidemiological approach. Online delivery of content predominated in continuing professional development training, whereas in undergraduate healthcare curriculum, delivery strategies varied. Overall, the process of embedding PA in HCPs’ curriculum and culture is ongoing. In addition to highlighting what is present within healthcare education, this study identifies further opportunities. Potential avenues include extending PA promotion into other workforces, including social workers.

## 1. Introduction

Getting healthcare professionals (HCPs) involved in the promotion of physical activity (PA) is one of the best investments to reduce the risks of inactivity and sedentary behaviour [1,2,3]. HCPs include physiotherapists, nurses, and general practitioners (GPs). In the UK, there are over 650,000 HCPs who are estimated to each see nearly half a million patients during their career [4]. These professionals are considered a trusted source of health-related information and guidance, meaning that their advice can widely influence PA levels. Evidence indicates that one in four people would be more active after the promotion of PA in healthcare services [5]. This would translate to 2.9 million less inactive adults in England. Yet, the involvement of HCPs in PA promotion is limited. Despite PA guidance from the National Institute for Health and Care Excellence, only 56% of medical schools in the UK teach the UK Chief Medical Officers’ recommendations and guidance on PA to future doctors [6,7]. Of further concern, nearly three quarters of GPs do not have PA conversations with patients [8], with 80% reporting being unfamiliar with the national PA guidelines [9]. Perceived barriers to discuss PA with patients include a lack of training, knowledge, confidence, and time [10,11,12].

In the light of the foregoing, upgrading the education and training of HCPs has become a public health priority in the UK. Here, ‘Making Every Contact Count’ (MECC) supports HCPs to talk with patients about PA during routine interactions. In parallel, a national programme called ‘Moving Healthcare Professionals programme’ (MHPP) was initiated in 2017 to provide evidence-based PA training and support resources for HCPs, capturing medical education that ranges from undergraduate study to continuing professional development (CPD) [13]. To date, more than 28,000 HCPs have been trained to promote PA by the MHPP network of PA clinical champions, and MHPP has been recognised as good practice internationally [13].

Yet, despite the ongoing success of these initiatives, there is room for doing better. Actions, however, need to be based on research knowledge. Before any improvements in the curricula of academic and vocational courses can be instigated, and so other countries or workforces can learn from UK training if desired, it is important to identify what and how HCPs have been taught to date. Therefore, this scoping review study was designed to present a broad overview of what the literature tells us about what PA promotion training have HCPs received in the UK and how has such content been delivered. It is hoped that the review will provide a knowledge base to critically reflect on what has been achieved so far and to set research, policy, and practice agendas for the future.

## 2. Materials and Methods

This scoping review adopted an established five-stage protocol by Arksey and O’Malley (2005), built upon by Levac, Colquhoun, and O’Brien (2010) [14,15]. It was also guided by the ‘Preferred Reporting Items for Systematic Reviews and Meta-analysis Protocols for Scoping Reviews’ checklist [16,17] (see Supplementary File S1).

### 2.1. First Stage: Aim and Research Questions

The aim of this paper was to determine what content is currently taught to HCPs and how this is delivered. The research questions were as follows: (1) What PA training content do HCPs currently receive? (2) How is PA training delivered to HCPs?

### 2.2. Second Step: Relevant Studies Identified

A three-step process was followed to identify relevant studies. First, seven electronic databases were consulted: Medline (Ovid Online, New York, NY, USA), SPORTDiscus (Ovid Technologies, New York, NY, USA), PsycINFO (American Psychological Association, Washington, DC, USA), EMBASE (Elsevier, Amsterdam, The Netherlands), CINAHL (EBSCO, Ipswich, UK), Cochrane Library (Cochrane, London, UK), and Web of Science (Clarivate Analytics, Philadelphia, PA, USA). A search of Grey literature included applying key search terms and analysing the first 100 search results on Google (Google LLC, Mountain View, CA, USA) and hand searching UK public health and sport organisation websites in line with Canadian Agency for Drugs and Technologies in Health ‘grey matters’ checklist [18]. Databases were searched for titles, abstracts, and keywords that contained the ‘Population Concept Context’ elements recommended for effective search criteria for scoping reviews (see Table S1, Supplementary File S2) [16]. Searches were conducted up to 21 March 2021. Appropriate truncations and wild cards were used to account for search term variation across databases, with support of Durham University library. A full electronic search strategy example for MEDLINE can be found in Table S2, Supplementary File S2. No limitation was set for year, and inclusion and exclusion criteria (see Table 1) were designed to be highly inclusive. Second, reference lists of included articles returned from the database search were scanned for relevant publications that the search strategy may have missed. Finally, authors of relevant studies from the database search were contacted as well as two stakeholders from Public Health England and Sport England, to request any further resources that they deemed relevant to the scope of this study. Interactions with stakeholders involved informal interviews and regular communication.

### 2.3. Third Step: Study Selection

**Figure 1:**
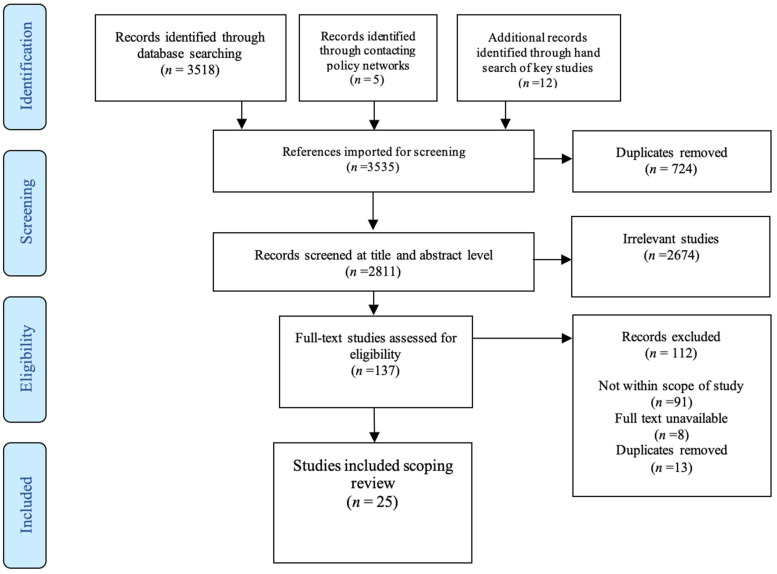
Study selection PRISMA flowchart.

The study selection process and reason for exclusion are presented in a flow diagram (Figure 1). The first author (JN) uploaded all 3535 studies to EndNote X9 software, where duplicates were removed. Next, titles, abstracts and keywords were screened, and full texts reviewed by first author, discussing inclusion and exclusion with BS and JM as part of an iterative process.

### 2.4. Fourth Step: Charting the Data

A data chart was constructed using Microsoft Excel to facilitate the ‘Population Concept Context’ information stated within each included study (see Supplementary File S3). The first author charted all study details, whilst the third author checked the accuracy of data extraction.

### 2.5. Fifth Step: Collating, Summarising and Reporting Results

Findings of this scoping review include descriptive numerical analysis that provided insight into the nature and distribution of included studies. In addition, a narrative summary of the evidence base articulates the findings through themes in relation to the studies’ research questions [14].

### 2.6. Patient and Public Involvement

Neither patients nor the public were involved in the design, reporting, or dissemination of this review.

## 3. Results

**Figure 2:**
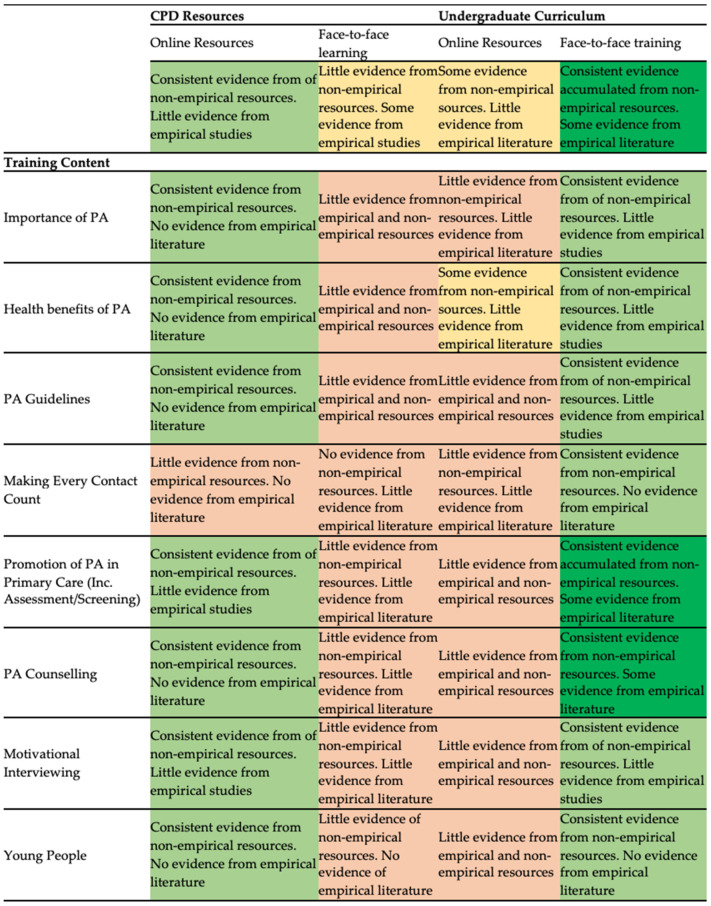

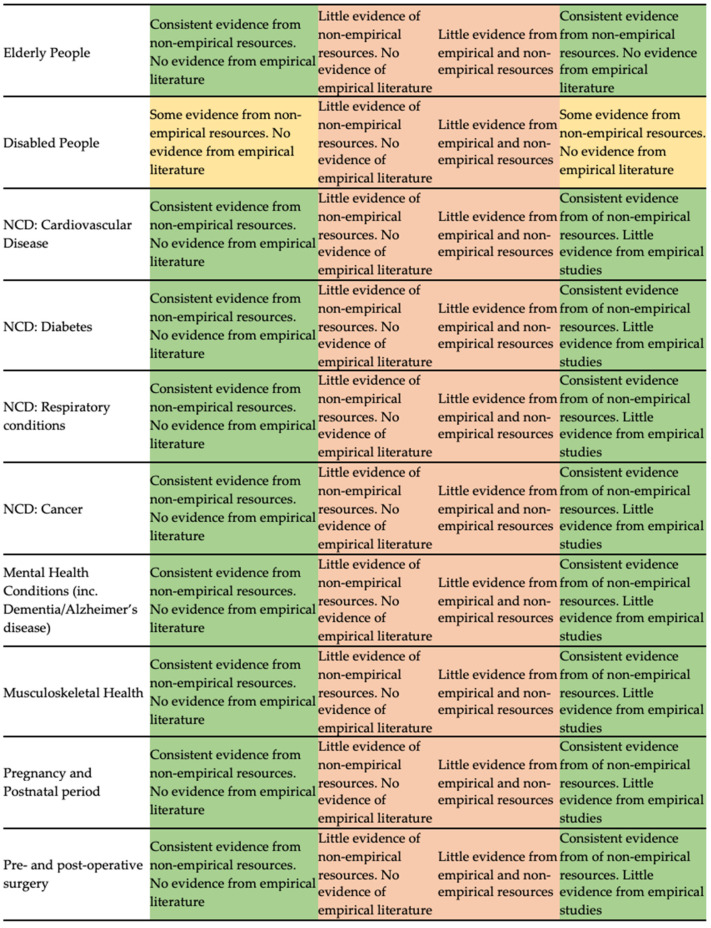

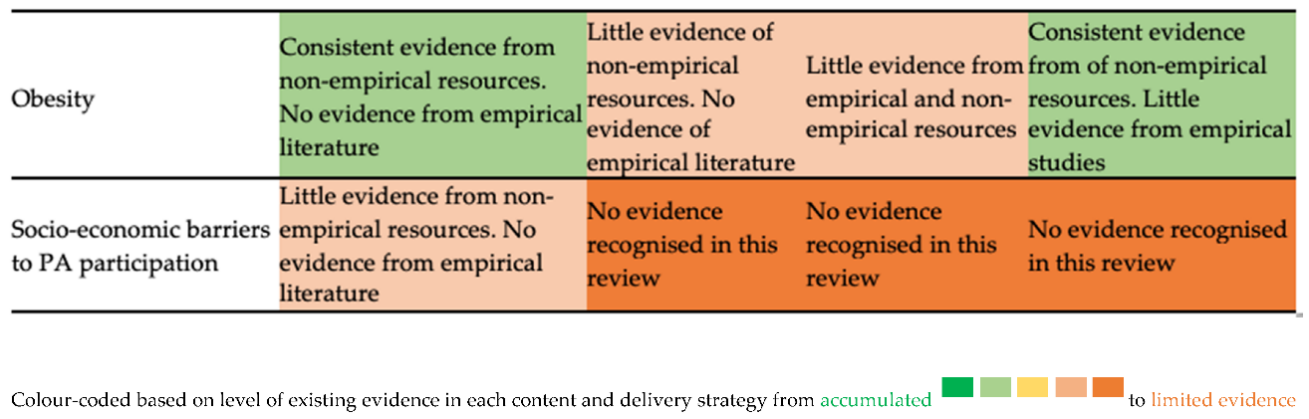
Colour-coded matrix based on quantity of evidence available of UK content and delivery strategies of PA promotion training to HCPs.

In total, 3535 references were identified for screening (database search (*n* = 3518); four academic and four policy networks were contacted from the UK (*n* = 5), and 12 studies were identified through hand searching reference lists and Grey literature reports). Following screening and removal of duplicates, 25 studies were included in the final analysis. Figure 1 presents a study selection flowchart, with summary of included studies detailed in Supplementary File S3. A comparative matrix was also used to display gaps in relation to dissemination and PA content training (Figure 2). The narrative summary of findings is organised according to our research questions. Following the review process, we considered the views of the stakeholders involved. Using the results as a prompt, we consulted two people working within Public Health England and Sport England who, as insiders involved in the MHPP, possess relevant knowledge that could be useful to add further insight.

### 3.1. What Physical Activity Training Content do HCPs Currently Receive?

The literature reviewed suggests that HCP CPD training and undergraduate curriculum share similar content across PA promotion training in the UK. Three themes capture the basic PA curricula content: health benefits of PA, PA promotion in healthcare, and PA and behaviour change.

#### 3.1.1. Health Benefits of PA

Both CPD training and undergraduate curriculum teach HCPs about the multiple benefits of PA. For example, students and professionals are taught about reductions in sedentary behaviour, reduced inflammation and oxidative stress to cells, weight loss, improved muscular adaptation, cognitive function, mental health, sleep quality, and management of diverse health conditions [18,19]. Content on the prevention and management of existing health conditions and NCDs also predominated [19,20,21,22,23,24,25,26,27,28,29,30,31]. Furthermore, UK PA guidelines and infographics encompassing different populations were key teaching resources (Table 2) [3,20,22,25,28,31,32].

#### 3.1.2. PA Promotion in Healthcare

CPD and undergraduates were trained on how to promote PA in healthcare environments. For example, they were trained on how to assess patient’s current PA behaviours within CPD training and undergraduate curriculum education [3,29,33,34,35]. In CPD training, content was delivered on how to use PA assessment questionnaires [29,31], PA capability assessments [34] and adding PA vital signs to patient health histories [19]. In undergraduate curriculum, the Frequency, Intensity, Type, Time (FITT) principle has been used to train HCPs in assessing current PA levels for patients [28]. The identification of risk factors when promoting PA to patients was an important content [19,20,27,29,33]. For example, HCPs were taught that contraindications include high blood pressure, unstable angina, unstable diabetes, and resting tachycardia [28]. Counselling and prescribing PA to patients was also taught [23,25,26,27,33]. For instance, trainees were encouraged to counsel PA using health condition consultation guides [3,30]. Furthermore, NHS frameworks such as MECC were also utilised [24,28,29,32].

One difference between CPD training and the undergraduate curriculum found in the literature was that strategies for how to guide HCP–patient conversations to promote PA were evident in CPD training. To do this, CPD training often used a 5As framework (Ask, Assess, Advise, Assist, Arrange) [19,20,22] and sought to encourage PA participation that is local, enjoyable, and practical [19]. Evidence of signposting to further PA resources and healthcare campaigns was included in both CPD training [19,20,29,30,31] and undergraduate curriculum [24,25], including the health campaigns Park Run (parkrun Limited, Twickenham, England) [24,30], The Daily Mile (The Daily Mile Foundation, Lyndhurst, England) [24,25], and Walk4Life (Walk Unlimited, Halifax, England) [29].

#### 3.1.3. PA and Behaviour Change

Content on PA and behaviour change was widely taught. One difference between CPD training and the undergraduate curriculum found in the literature is that, in CPD training, behaviour change models such as the ‘Capability, Opportunity, Motivation, Behaviour’ (COM-B) model were used [20,22,29]. In contrast, behaviour change strategies such as the motivational interview [20,26,29,36,37] were used in both education levels (Table 3). To illustrate this, training content for motivational interviewing in one CPD training resource included reflective listening, open-ended questions, and linking discussions to individual patient goals [20]. Often, CPD training focused on increasing patient capability to change via self-monitoring, goal setting, and social support [29]. Patient case studies were also employed to teach how to incorporate PA behaviour change into consultations with patients [19,20,26,29,30,31].

Despite this, one concern raised by stakeholders in relation to MHPP was the lack of focus on the patient voice: ‘because the programme is so focused on the HCP, the patient voice in that is difficult to find sometimes because it is so much further away from the intervention itself’. CPD training content looks to remedy this through patient stories from Sport England’s ‘We Are Undefeatable’ campaign [38]. Stakeholders perceived that this campaign provides lived experience content through stories for HCPs to gain confidence and competence in the importance of promoting PA to patients by ‘using the power of people’s stories about how their HCP helped them get active’. Often, these stories talk about diverse social determinants of PA behaviour and health, broadly defined as ‘the conditions in which people are born, grow, live, work, and age’ [39]. With exceptions [30,31], important determinants such as the socio-economic barriers of PA are largely overlooked in current training (see Figure 2).

### 3.2. How Is PA Training Delivered to HCPs?

PA promotion content was delivered in CPD training and undergraduate curricula in many ways via online or e-learning and face-to-face learning.

#### 3.2.1. E-Learning

E-learning has been adopted as it offers learners control over content, learning sequence, pace of learning, time, and often media, allowing them to tailor their experiences to meet their personal goals [40]. ‘BMJ Learning’ makes up part of the e-learning aspect of the MHPP [41], providing HCPs with over 4.5 h of PA promotion content across the lifespan and health conditions [19]. Knowledge on PA was disseminated to HCPs via nine online modules (see Table 3) that included text, videos, patient case studies, signposting to further resources, and online assessments for HCPs to complete. Another programme that delivered PA knowledge to HCPs online as part of the MHPP was ‘E-learning for Healthcare’ [20], which delivered training over 11 online modules that shared similar PA promotion content and designs as ‘BMJ Learning’. A third online training programme highlighted in the literature was ‘Motivate2Move’ [29], which consists of 18 PA chapters containing information alongside a factsheet for HCPs to use as resources in working practice. This programme also signposted both staff and patients to additional on PA guidance [29].

A fourth key resource identified in the review was ‘enabling and encouraging PA e-learning’ by Public Health Scotland [31]. This programme shared similarities to the three aforementioned training programmes, whereby information was provided online in chapters, including final assessment as in ‘BMJ Learning’ and ‘E-learning for Healthcare’ [31]. Finally, ‘Moving Medicine’ was regarded a major online consultation resource for HCPs. How PA was taught here differed to all other resources, with evidence-based consultation guides available for HCPs to guide 1 min, 5 min, or longer consultations with patients to promote PA across age ranges and relevant health conditions. ‘Moving Medicine’ also provide patient story videos, including Sport England’s ‘We Are Undefeatable’ campaign, to promote PA in response to health conditions [30]. Undergraduate curriculum training found in the literature also showed evidence of using online web portals for house training, such as slide set resource ‘Movement for Movement’, to supplement and embed PA training within current undergraduate HCP curriculum [3,21,42]. In the undergraduate HCP curriculum, podcasts were an example of how PA content was delivered to HCPs via alternative online platforms [24,25]. Overall, evidence supports that the online delivery of CPD training is effective [41].

#### 3.2.2. Face-To-Face Learning

CPD training and undergraduate curriculum training for HCPs differed in approaches to face-to-face training. In CPD training, face-to-face teaching came in protected learning time, delivered by fellow HCPs [36]. As part of the MHPP, designated HCP ‘Clinical Champions’ delivered CPD training to other HCPs via slide sets during protected learning time, vocational training schemes, and conferences. Within this training, ‘Clinical Champions’ would signpost additional CPD training resources, such as ‘Moving Medicine’, to complement face-to-face training [35]. Further face-to-face CPD training found in the literature incorporated training in how to deliver PA promotion using PA assessment tools, including tuition on using a PA assessment calculator and motivational interviewing [34], and face-to-face training on how to use PA clinical advice pads [36].

In contrast, the undergraduate curriculum provided face-to-face training by embedding PA promotion into existing healthcare modules in a spiral approach [27], delivered by curriculum teachers as opposed to peer-to-peer training provided for current HCPs. Examples of a spiral curricula approach whereby schools of medicine or health can tailor PA training resources (such as ‘Movement for Movement’ [27]) to their patient needs was evident within the literature. For example, an example in the literature identified that reported PA resources were taught face-to-face in year one of study and specific resources of PA promotion in relation to NCDs in year two, with online resources used to complement PA promotion training [21]. Another example of a spiral curricula approach was one case study whereby first-year students received two face-to-face lectures on the links between PA and health, and the role of medical professionals in assessing and counselling PA, respectively, whilst having access to additional online content including ‘Movement for Movement’ slides, ‘Moving Medicine, and monthly blogs on PA [3]. Further evidence of face-to-face teaching within the undergraduate curriculum was present in one study that integrated PA behaviour change training into four lectures spread across three years of exercise medicine curricula [43]. PA training was embedded within existing health topics as a prevention and disease management strategy [33].

## 4. Discussion—What Now? What Next? Discussing Results, Identifying Gaps, and Providing Directions

Embedding PA promotion in healthcare can be a very successful strategy to improve population health and wellbeing. Success, however, is conditional to HCPs and their knowledge on how best to inspire and support individuals to be active. It is therefore crucial to provide training opportunities to increase the skills, competence and confidence of HCPs. In the UK, training on how to raise and promote PA with patients has been introduced both into the university core curriculum teaching and CPD training. How, though, this has been achieved? What might be learnt from the available literature? We offer the following takeaway points from the scoping review.

First, we have learned from the literature that no standardised approach to how PA training has been adopted in the UK. Standardisation is achieved by applying a clear set of guidelines and best practices. This would help policy and decision makers be able to better evaluate, measure and predict teaching against the required outcomes, as well as to better understand and communicate the training structure to key local, national, and international stakeholders. Such benefits acknowledged that a standardised framework for PA training can have negative side effects. For example, standardisation can lead to prioritising whatever is easier to measure and to overlook the learning process. HCPs could become mere ‘consumers of pre-packed education’ [44] designed by expert groups who often may not teach the programs they design and reproduced by teachers devoid of pedagogical autonomy. Pedagogical alternatives include ‘curriculum work as craft’ [45] or a ‘possibility knowledge’ framework [46]. The question of what we gain and lose with total, partial or no standardisation requires further discussion moving forward.

Second, UK training content is largely underpinned by an epidemiological approach. The relevance of PA epidemiology is unquestionable [47]. However, some dimensions involved in PA promotion cannot be tackled just through epidemiological knowledge, regardless of how exact this is. In navigating the intricacies of PA promotion, interdisciplinary content is essential [48]. For example, it is important HCPs learn about the social determinants of health and how they affect people’s ability to practice healthy behaviours. In specialised journals such as ‘*Academic Medicine’* and ‘*Medical Teacher’*, the significant role of different approaches in health professions education, such as the medical humanities approach, is also highlighted [49,50]. Patients are human beings who often engage in PA for human reasons that cannot be predicted or explained through causal links, but rather apprehended through reflective practice. In short, balancing the scientific with the humanistic will position HCPs to have more productive PA conversations with patients.

Third, although our review did not intend to compare face-to-face learning with e-learning, exploring the advantages and drawbacks of these teaching methods and how they affect the learning experience of HCPs will be important to improve training programmes. Interestingly, evidence shows that most medical students view e-learning as enjoyable and effective yet do not see it replacing conventional classrooms [51]. Employing blended learning can thus be a fruitful third area to explore. Currently, the MHPP is the only training programme in the UK that complements e-learning with peer-to-peer training via Clinical Champions training. Peer-mentoring activities have been suggested as a key tenet in addressing other influential determinants in changing the behaviour of a HCP to promote PA to patients that go beyond knowledge competencies, potentially addressing influential determinants such as self-efficacy, competence and fear [12]. Stakeholders suggested that the peer-to-peer aspects of the training helped improve HCP competency in delivering PA guidance to patients as ‘*GPs believe other GPs, so its who those trusted messengers are ... It’s actually the narrative that the Clinical Champions bring with it and the personal stories that they tell of: I did this, and it had this impact on my patient’.* Blended learning can operate through other directions. For example, evidence suggests that the flipped classroom approach in education of health professionals has a significant potential, with students expressing high levels of satisfaction [52]. A flipped classroom is a blended learning modality where students are introduced to new content at home and complete their homework during class time. Further research is needed to establish its potential within PA promotion training.

Fourth, content has been mainly delivered through the Knowledge Deficit Model, a model of communicating that ‘emphasises the repetition of emotionless objectively sterile information to increase understanding’ [53]. One way of addressing the shortcomings of this model is through disseminating storied content. ‘Memorable’ educators tell stories [54] and, as we know from narrative medicine, stories themselves teach. For example, a study showed that adults with spinal cord injury and HCPs working with them ‘envisaged that the stories might be useful as professional training resources or pedagogical resources that can be used to teach people about physical activity in authentic and engaging ways’ [55] (p. 309). As highlighted, peer-to-peer training offers proper conditions for sharing anecdotes and stories about personal experience and knowledge.

Fifth, a barrier to delivering PA training within the undergraduate curriculum is the concern that the established curricula are already full, with little room for additional material [24]. That barrier becomes greater when reports that HCPs consider PA to be beyond their remit and less important than other health promotion activities such as smoking cessation [56]—even though PA can be used for stopping smoking—are factored in. One report highlighted the incorporation of PA assessment and examination opportunities provided to medical students as a way of embedding PA into current curriculum. At present, three medical schools have agreed to identify best-answer exam questions and to share their outputs across the Medical School Council Assessment Alliance [24]. Moving forward, that barrier needs tackling and creative solutions need to be invoked.

Finally, by focusing on HCPs in this review, it is vital to stress that PA promotion should not be reduced to that workforce only. Healthcare is an important investment to support the achievement of targets to reduce physical activity. However, we must expand our investments into other workforces. We need to expand training beyond just HCPs if we are to reduce physical inactivity because it would be dangerous to ‘put all our eggs in one basket’—especially in light of the increasing demands placed on HCPs, including during and after global pandemics. Recent literature [57,58] and stakeholders confirmed such concerns: ‘*We hear a lot from HCPs: it should not just be us trained in this’.* We also need to expand PA promotion into other workforces because there could be untapped credible, trustworthy, and wide-reaching PA messengers to help ‘make every contact count’. For example, in relation to what works for PA, there is evidence that disabled people consider social workers to be excellent messengers of PA [59]. At the same time, social workers have a strong enthusiasm and are willing to promote physical activity. However, they have identified that to achieve this they need education and training in PA promotion [60]. Work is underway to achieve this.

Of course, such work must be supported by professional regulatory organisations. It is also important that physical activity societies such as the *International Society of Physical Activity and Health* (ISPAH) expand calls for action to reduce physical inactivity by including other workforces into policy and investments. Training HCPs in PA promotion is vital. However, we cannot leave all the work to them when we not only know that their time with patients is increasingly challenged, but also that other workforces have been identified as useful and having a wide reach when it comes to PA promotion. Let us help ‘Make every contact count’ by learning from what is taught and how in HCP PA training. We can then use appropriate knowledge from that learning to help train and educate other workforces such as social workers. The plurality of appropriate messenger groups seems to be a promising opportunity to improve PA levels moving forward.

## Figures and Tables

**Table 1:** Inclusion and exclusion search criteria for electronic database search.

Inclusion Criteria	Exclusion Criteria
UK-based research articles	Abstracts without full text
Research conducted in any student or HCP populations	Articles focusing on the behaviour change of the participant not within the scope of this review (e.g., investigating the PA behaviours of medical students)
Articles published in peer-reviewed or Grey literature	Articles that did not include the concept of what PA training was included within participant training
Articles published in English	Articles that did not include the context of how PA training was delivered to participants
Research designs including, but not limited to; qualitative, natural experiment with pre-post measures, content analysis, systematic or non-systematic reviews, commentary, theory, summary, policy, or practice papers	Non-UK based research articles
Articles stating the context and concept of the applied training delivered to the relevant participant	Articles not in English language
Training resources highlighted within literature	

**Table 2:** E-learning and online training resources stated within included studies.

Curricula Content	BMJ E-Learning (MHPP) [20]	E-Learning for Healthcare (MHPP) [19]	Movementfor Movement (Based on ERASMUS How-to Guide) [28]	Motivate2Move [29]	Moving Medicine [30]	Public Health Scotland [31]
Importance of PA	✓	✓	✓	✓	✓	✓
Health benefits of PA	✓	✓	✓	✓	✓	✓
PA Guidelines	✓	✓	✓	✓	✓	✓
Promotion of PA in Primary Care	✓	✓	✓	✓	✓	✓
PA Counselling	✓	✓	✓	✓	✓	✓
Motivational Interviewing	✓	✓	✓	✓	✓	✓
Young People	✓	✓	✓	✓	✓	✓
Elderly People	✓	✓	✓	✓	✓	✓
Disabled People	✓ *	✓ *	✓	✓	✓	✓
NCD: Cardiovascular Disease	✓	✓	✓	✓	✓	✕
NCD: Diabetes	✓	✓	✓	✓	✓	✕
NCD: Cancer	✓	✓	✓	✓	✓	✕
Mental Health Conditions (inc. Dementia/Alzheimer’s disease)	✓	✓	✓	✓	✓	✕
Musculoskeletal Health	✓	✓	✓	✓	✓	✕
Pregnancy and Postnatal period	✕	✓	✓	✓	✓	✓
Socio-economic barriers to PA participation	✕	✕	_ **	✕	✓	✓

* = PA guidelines infographic present. ** = unspecified in training contents.

**Table 3:** Contents and dissemination strategy for CPD training and undergraduate curricula resources identified in the literature.

Resource	Movement for Movement [28]	BMJ Learning [19]	E-Learning for Healthcare [20]	Motivate2Move [29]	Public Health Scotland [31]	Moving Medicine [30]
Who does this training target?	Undergraduate curriculum	CPD training	CPD training	CPD training	CPD training	CPD training
How training is disseminated?	Slide-set resources to be embedded in Undergraduate education	E-learning	E-Learning	Online resources and factsheets	E-learning	Online consultation guide
Training content chapters	Leadership on PA in health and wellbeing	The importance of PA	Introduction to PA	UK PA guidelines	What is PA?	PA and patient age type
	World Health Organization (WHO) and UK PA guidelines and recommendations	How does PA produce health benefits?	Promoting PA in primary care	All-cause mortality (longevity)	UK Chief Medical Officers’ (CMO) guidelines	PA and stroke
	MECC	The health benefits of PA: cancer	Children and young people: Being active	Cancer	How active are we?	PA and amputee
	Brief interventions and making every influence matter	The health benefits of PA: Diabetes	Older adults: Being active	PA and cardiovascular health	Benefits of PA	PA and cancer
	Cancer and exercise	The health benefits of PA: osteoarthritis and lower back pain	Cardiovascular conditions: Being active	Chronic kidney disease	Key Messages	PA and Chronic Obstructive Pulmonary Disease (COPD)
	CVD, exercise and cardiac rehabilitation	The health benefits of PA: cardiovascular disease	Type 2 Diabetes: Being active	PA & mental health	National PA pathway	PA and Dementia
	T2 Diabetes and exercise	The health benefits of PA: respiratory disease	Cancer conditions: Being active	Neurological disorders	Raising PA	PA and Depression
	Mental health and exercise	The health benefits of PA: depression, anxiety, sleep, and dementia	Mental health: Being active	Obesity	Screening for activity	PA and inflammatory rheumatic disease
	Osteoporosis, sarcopenia and exercise	The health benefits of PA: promoting PA in primary care	Musculoskeletal health: Being active	PA and pregnancy	Delivering PA advice	PA and ischaemic heart disease
	Fall and exercise		Pregnancy and postnatal period: Being active	PA and respiratory disease	Signposting and referral	PA and musculoskeletal pain
	COPD, exercise and pulmonary rehabilitation		Motivational Interviewing video	Sedentary behaviour	Follow up and review	PA and primary prevention
	Surgery and exercise			Supporting people to change their health behaviour	Key resources	PA and type 2 Diabetes
	Hypertension and exercise			Starting to exercise		PA and pregnancy
	Stroke, exercise and rehabilitation			Resources for primary care		Support and local activities
	Deconditioning and exercise					Why movement matters
	Obesity and exercise					Consensus statement
	Rheumatoid arthritis and exercise					Case studies ‘We Are Undefeatable’
	Pregnancy pre/post and exercise					
	Chronic kidney disease and exercise					
	Environment, health and PA					
	Intellectual disability and PA					
	School for change agency and leadership					
	Leadership on PA in health and wellbeing					

## Data Availability

See Supplementary File S3 for data chart of included studies.

## Supplementary Materials

The following are available online at https://www.mdpi.com/article/10.3390/ijerph18136701/s1, Supplementary File S1: PRISMA checklist; Supplementary File S2: Search terms; Supplementary File S3: Charted studies.
