# Supplementary material for: Training Healthcare Professionals on How to Promote Physical Activity in the UK: A Scoping Review of Current Trends and Future Opportunities
Source: Int J Environ Res Public Health. 2021 Jun 22;18(13):6701. doi: 10.3390/ijerph18136701 (PMC8297298; doi:10.3390/ijerph18136701)
Supplement: Supplementary file 1 [file ijerph-18-06701-s001.zip › Supplementary File 2.pdf]

Supplementary File 2: Search Terms and Database Strategy

Table S1: Search terms used in database search

**Population**	**Concept**	**Context**
Healthcare Professional*	Physical activity	education
Health Professional*	Physical fitness	e-learning
Healthcare worker*	Health promotion	eLearning
Clinician	Physical Exercise*	curricula
Consultant		curriculum
Doctor		undergraduate
General practitioner		postgraduate
physician		Continuous professional development
Medical professional*		CPD
Medical student*		training
Nurses/Nursing/nurse		
Occupational Therap*		
Physiotherap*		
Allied Health Professional		
Social Work*		

Table S2: Example search strategy (MEDLINE)

#	**Searches**
1	((Health Worker or Health$ Worker or Health Professional or Healthcare Professional or Medic$ student or Medic$ undergraduate or Medic$ postgraduate or Nurse or Nursing or Nurses or Occupational Therapist or Physiotherap$ or Allied Health Professional$ or social work or Doctor or Physician or Clinician or Consultant) and ((Physical adj (Activity or Fitness or Exercise)) or Health Promotion) and ((university or undergraduate or postgraduate or professional development or CPD) and (education or training or curricul$ or elearning or e-learning))).ab.
2	limit 1 to english language
3	((Health Worker or Health$ Worker or Health Professional or Healthcare Professional or Medic$ student or Medic$ undergraduate or Medic$ postgraduate or Nurse or Nursing or Nurses or Occupational Therapist or Physiotherap$ or Allied Health Professional$ or social work or Doctor or Physician or Clinician or Consultant) and ((Physical adj (Activity or Fitness or Exercise)) or Health Promotion) and ((university or

	undergraduate or postgraduate or professional development or CPD) and (education or training or curricul$ or elearning or e-learning))).ti.
4	limit 3 to english language
5	((Health Worker or Health$ Worker or Health Professional or Healthcare Professional or Medic$ student or Medic$ undergraduate or Medic$ postgraduate or Nurse or Nursing or Nurses or Occupational Therapist or Physiotherap$ or Allied Health Professional$ or social work or Doctor or Physician or Clinician or Consultant) and ((Physical adj (Activity or Fitness or Exercise)) or Health Promotion) and ((university or undergraduate or postgraduate or professional development or CPD) and (education or training or curricul$ or elearning or e-learning))).kf.
6	limit 5 to english language
